# Education in primary care: personal tuition, teamwork and lots of patients

**DOI:** 10.5116/ijme.6171.27bd

**Published:** 2021-10-30

**Authors:** Simon Gay

**Affiliations:** 1 School of Medicine, University of Leicester, Leicester, UK

**Keywords:** Education, primary care, personal tuition, teamwork

## Introduction

The education of health professionals in primary care has expanded substantially during the three decades since I first qualified as a family medicine doctor, or general practitioner (GP), as members of my clinical discipline are more commonly known in the UK.

I can recall visiting GPs in their place of work in South London when I was an undergraduate medical student, having been given very little guidance on what I was meant to learn before I attended, and simply being sat in a corner as a passive observer of healthcare when I arrived. I did not see any other students from either my own or any other health profession, nor did I see any qualified doctors in training to become GPs.

However, at that time, the very fact that I was given any clinical placement in a community medicine setting represented progress as medical education moved slowly away from the Flexnerian^1^ concept of a preclinical bioscience phase of education followed by an apprenticeship phase of clinical learning conducted entirely within the walls of teaching hospitals towards more integrated, learner-centred curricula with the inclusion of community healthcare experience. This shift was beautifully encapsulated by Harden and colleagues’ Spices Model,^2^ which challenged curriculum designers to decide where on a set of spectra they wished their curriculum to lie.

But what has happened since? Well, the number of doctors training to become GPs, though still not at the level needed in the UK, has increased. The number of doctors graduating from UK medical schools has also increased from 4432 in the year 2000 to 8730 in 2021,^3^ and the proportion of time spent in primary care by medical students has increased “from <1.0% of clinical teaching in 1968 to 13.0% by 2008; (though) since then, the percentage has plateaued”.^4^ Furthermore, other healthcare professions such as pharmacy, nursing and physician associates are now also spending some of their time in training in primary care.

So, what is it about primary care that makes it such a good place to learn about healthcare? There are very many features of education in primary care that could be offered in response to this question, and I can only cover some of them in this article, but my answer starts by quoting the eminent Australian GP and medical educationalist, Richard Hays, who observed that in primary care…

“…multi-professional teamwork is well established, and patients are plentiful…”^5^

…so, let’s consider each of those in turn. Whilst more recently multidisciplinary teams have become much more common in secondary care settings across the world, they have been a central feature of clinical practice in primary care for very many years. Working practices and the roles of the various members of the team are well established, and membership of those teams is often relatively stable over long periods of time. Such stability promotes trust and mutual understanding and strongly contributes to the creation of a positive learning environment where learners of any primary care related discipline can be nurtured and developed. Furthermore, the value of the learning about teamwork gained in primary care is not confined to that setting but is also largely transferable to the modern multidisciplinary teams now also found in secondary care. Indeed, for some learners who choose to pursue a career in some secondary care specialities, their undergraduate experience of primary care may be their only time spent in a community setting for the entirety of their training, making this experience not only important but essential if they are to properly understand the wider context of healthcare in which they will go on to provide their secondary care service.

Moving on, why is having a plentiful supply of patients so educationally important? My response to this question has to start with one of the most often cited William Osler quotations…

“He who studies medicine without books sails an uncharted sea, he who studies medicine without patients does not go to sea at all.” ^6^

Some aspects of this statement have not stood the test of time well. For instance, in many medical schools across the world, female students now outnumber male students at entry. Similarly, whilst textbooks are still relevant, students are now more likely to study an electronic version of any given text rather than the printed book, they are also more likely to watch a video recording of a lecture than attend the lecture itself and regularly participate in some form of self-directed online learning. However, what has not changed is that students still cannot truly learn to practise medicine without consulting with patients.

Patients are usually generous with their time, permitting learners to practise both their history taking and examination skills upon them, sharing the most private details of their health story and exhibiting the utmost patience in the face of what can occasionally become quite a laborious process from the patient’s perspective. Yet, for the learner, such experience is priceless.

“Evidence-based medicine is the integration of best (current) research evidence with clinical expertise and patient values…” ^7^

…and a key element of this triad is the patient. Until the learner can apply their prior learning properly to the patient in front of them, they may know much but can actually do very little of any use with that knowledge. The ability to be truly patient-centred when practising medicine is most quickly learned by seeing lots and lots of patients, and the more our students can come to appreciate any given consultation from the patient’s perspective, the more insightful and efficient their consultations will become.

However, it is not only the number of patients available that makes primary care such fertile ground for learning. In many health economies in recent years, there has been a shift of healthcare from secondary care out into the community. Patients who do require secondary care management are often only in the hospital for very short periods of time and are only very briefly, if at all, available to students for their learning before they leave the hospital again. It can therefore be much easier for students to encounter hospital-based care by becoming aware of a patient’s trajectory toward hospital before the hospital component of care even takes place and then, with the patient’s consent, accompanying the patient on their journey through secondary care. This is just one of the many benefits of educating medical students in educational structures known as longitudinal integrated clerkships (LICs).^8^ LICs most often occur in primary care, but some also occur in secondary care; and this also relates to another important educational feature of primary care - the sense of continuity offered to both patients and learners. We know continuity has beneficial consequences for patients,^9^ and as LICs again demonstrate, continuity has beneficial consequences for learners, too.^10^

Another important feature of the patients found in primary care is that they usually present to the clinician in an unfiltered way. That is to say, even when the patient is known to the clinician, the reason for them presenting on that specific occasion is usually not known. This is of particular value to novice learners as every presentation offers them the opportunity to be the first person to take a medical history from the patient, without any significant clues about what may be the matter with the patient on this occasion. Uncorrupted patient stories offered during first presentations to healthcare are superb opportunities for learners to hone their consultation skills. Once the patient has been questioned about their story once they will never tell it in quite the same way again, often subliminally realising which features are of most interest to the clinician and unwittingly modifying their presentation to others they subsequently speak to about the issue at hand as a result.

This precious unfiltered nature of primary care consultations is of particular value to learners in their acquisition of clinical reasoning skills and lends itself very well to the activity Ericsson called deliberate practice – “strategic, focused, goal-orientated activities aimed at improving performance”.^11^ However, in addition to exposure to unfiltered patients, there are also a number of other features of primary care that make it an especially good environment for learners to gain deliberate practice. The easy access to a relatively constant stream of patients, for example, enables multiple repetitions of consultations to be performed in a relatively short space of time, with focussed feedback provided between repetitions in a timely and constructive manner.

Finally, the subject of high-quality feedback brings me on to yet another key component of primary care education, the clinical education supervisor. The presence of a single overarching clinical education supervisor for the entire length of a clinical placement permits a supportive educational relationship to develop over time, and so offers ample opportunity to capture the trust and respect such a relationship should embody. Furthermore, it allows the supervisor to offer sequential feedback to help the learner to achieve incremental improvement, with both learner and supervisor using each session to build upon the last. In this way, the learner can be situated in a regularly updated and refreshed zone of proximal development^12^ whilst the supervisor provides appropriate scaffolding^13^ to aid their forward momentum.

These features also support the learner to move beyond the artificial finishing line of diagnosis, a finishing line that is so often promoted within the published clinical reasoning literature that one might be forgiven for thinking healthcare stops at that point. Medical students and doctors in training can benefit greatly from learning the value of seeing a patient more than once, learning to formulate their own management plans and committing to them without being able to “play the student card”^14^ (not being able to opt out at the decision-making point), and learning the value of the doctor as drug.^15^

In conclusion, therefore, primary care has a great deal to offer the education and training of healthcare professionals. The knowledge, experience and fertile learning environment found in primary care are superb resources available to clinical education in many countries around the globe and curriculum designers would be wise to take advantage of that if they are not already doing so.

### Conflict of Interest

The author declares that they have no conflict of interest.
